# Pharmacological Isolation of Cognitive Components Influencing the Pupillary Light Reflex

**DOI:** 10.1155/2015/179542

**Published:** 2015-05-24

**Authors:** Stuart R. Steinhauer, Ruth Condray, Misha L. Pless

**Affiliations:** ^1^Biometrics Research Program, 151R, VA Pittsburgh Healthcare System, University Drive C, Pittsburgh, PA 15240, USA; ^2^Department of Psychiatry, University of Pittsburgh School of Medicine, Pittsburgh, PA 15260, USA; ^3^Leitender Arzt Neuroophthalmologie und Neurologie Augenklinik, Zentrum für Neurologie und Neurorehabilitation, Luzerner Kantonsspital, 6000 Luzern 16, Switzerland

## Abstract

Cognitive operations can be detected by reduction of the pupillary light response. Neurophysiological pathways mediating this reduction have not been distinguished. We utilized selective blockade of pupillary sphincter or dilator muscles to isolate parasympathetic or sympathetic activity during cognition, without modifying central processes. Pupil diameter was measured during the light reaction in 29 normal adults under three processing levels: No Task, during an easy task (Add 1), or a difficult task (Subtract 7). At three separate sessions, the pupil was treated with placebo, tropicamide (blocking the muscarinic sphincter receptor), or dapiprazole (blocking the adrenergic dilator receptor). With placebo, pupil diameter increased with increasing task difficulty. The light reaction was reduced only in the Subtract 7 condition. Dapiprazole (which decreased overall diameter) showed similar task-related changes in diameter and light reflex as for placebo. Following tropicamide (which increased overall diameter), there was a further increase in diameter only in the difficult task. Findings suggest two separate inhibitory components at the parasympathetic oculomotor center. Changes in baseline diameter are likely related to reticular activation. Inhibition of the light reaction in the difficult task is likely associated with cortical afferents. Sustained sympathetic activity also was present during the difficult task.

## 1. Introduction

Activation of mental and emotional processes is accompanied by changes in pupil diameter [1–3]. While most often assessed in terms of dilation of the pupil during experimental paradigms, another index of central processing has been observed as reduction in the amplitude of the light response. Psychosensory attenuation of the light reflex has been observed most often during emotional arousal, including anticipation of aversive stimuli [3, 4]. The light reflex is reduced in patients with anxiety disorders relative to controls [5], and in patients with schizophrenia [6], though the latter findings are less consistent [7]. Viewing of pictures matched for brightness indicated that the light reaction was significantly attenuated when comparing affective stimuli (violent or erotic scenes) as compared to neutral scenes [8]. Motor activity also has been demonstrated to reduce the extent of the light reaction [9].

Reduction of light reflex amplitude during cognitive operations also has been demonstrated. Subjects required to predict the occurrence of equiprobable sound and light stimuli exhibited smaller light reactions than under conditions of certainty [10]. Using a mathematical challenge task during presentation of light stimuli, initial pupil diameter increased, and the amplitude of constriction to light decreased [11]. Within the same session, a nonchallenging mathematical task that also required verbalization evoked an intermediate increase in overall diameter, but no reduction of the light reaction.

The central mechanisms that contribute to these changes can be identified as emanating from specific sympathetic or parasympathetic pathways [12]. Increases in diameter are produced by the sympathetic branch of the autonomic nervous system by integrated activity of the posterior hypothalamus, which projects to the dilator muscles of the iris. Decreased activity of this pathway is likely to decrease overall diameter but should not affect the light reaction directly. The parasympathetic system involves the Edinger-Westphal (E-W) complex in the oculomotor nucleus. This is the central motor center for the constrictor muscles of the iris. Inhibition of tonic activity of this region would lead to relaxation of the muscles, also resulting in dilation. In addition, interference with phasic activation would result in reduced responsivity to light stimuli. A variety of inhibitory inputs, including ascending reticular and descending cortical systems, act to inhibit E-W activity [3, 13]. It was suggested by Steinhauer et al. [11] that changes in overall diameter during cognitive operations were likely related to reticular activation but that reduction in the light reflex amplitude was associated with cortical activation. At the least, more than one aspect of these changes would need to be demonstrated to be associated with inhibitory activity emanating from the E-W complex.

Using topical administration of agents that diffuse through the cornea to the iris, it is possible to isolate activity associated with sympathetic or parasympathetic activity. Blockade of the pupillary dilator muscles can be accomplished using dapiprazole, an *α*-1 adrenergic antagonist [14, 15], such that any remaining motility of the pupil is attributable to changes in the parasympathetic pathway. Thus, Giakoumaki and colleagues [15] were able to demonstrate that inhibition of light reflex by fear anticipation was mediated by the parasympathetic pathway. Conversely, blockade of the muscarinic receptor of the pupillary constrictor muscles can be obtained with tropicamide, so that remaining pupillary changes are attributable to sympathetic activity. These agents are commonly employed during ophthalmologic examination, have a short (~30 min) time to reach maximum efficacy, and are metabolized within a few hours. By placing drops in a single eye, the other eye may be used for comparison of the peripheral effect. A particular advantage of this approach is that no interference occurs with central processing activities. That is, all central sympathetic and parasympathetic activity remains intact at all times. Using this approach, we examined task-related effects on tonic pupil diameter, demonstrating that both inhibition of the parasympathetic and sympathetic activity separately contributed to enlargement of pupil diameter as task difficulty increased [16]. This procedure was employed in the current study to determine the extent to which changes in overall pupil diameter and constriction to light could be localized to modulation in sympathetic or parasympathetic activity during performance of a cognitive challenge.

## 2. Materials and Methods

### 2.1. Subjects

Subjects were 29 volunteers (13 females) aged 21–42 years (mean = 28.9, s.d. = 6.2) with an educational level ranging from 12–21 years (mean 16.2). Five subjects were African American and 24 were Caucasian. Two subjects were left-handed. All subjects signed informed consent and were screened to exclude history of major medical disorder or DSM-IV AXIS I psychiatric disorder. The protocol was approved by the Institutional Review Boards of the VA Pittsburgh Healthcare System and the University of Pittsburgh. The subjects had been included in the prior study of the light reaction and task difficulty [11].

Narrow angle refers to a small physiological angle between the iris and the trabecular meshwork, which is the drain for intraocular fluid. In persons with narrow angle, there is risk of acute narrow angle glaucoma when the pupil is dilated, because the iris tissue blocks the drainage process. Ophthalmologic screenings were conducted to exclude the presence of narrow angle, since use of mydriatics such as tropicamide can lead to increased intraocular pressure in the presence of narrow angle when the iris is artificially dilated. No subjects were excluded on this basis, and no other ophthalmologic problems (other than correctable vision) were observed in any of the subjects.

### 2.2. Pharmacological Procedures

In addition to a base recording session involving no instillation of drugs (reported previously for the same subjects in [11]), each subject participated in three additional sessions involving placing of drops in the eye, which are the findings presented in the present report. At one session, ophthalmologic saline (Muro 128 2%) was employed as a placebo condition. At a second session, 1.0% tropicamide (Mydriacyl) was used to provide temporary blockade of the sphincter muscle. At a third session, 0.5% dapiprazole HCl (Rev-Eyes) was used to provide temporary blockade of the dilator muscle. (Dapiprazole HCl (Rev-Eyes) is not currently commercially available in the United States. A 2013 FDA notice indicates that it was not withdrawn from sale for reasons of safety or effectiveness.) The concentrations of tropicamide and dapiprazole are those normally employed in the ophthalmology clinic and cause temporary blockade for up to several hours, though not necessarily complete abolition of motility. Dapiprazole also tends to produce transient reddening of the sclera. The order of drug administration was randomized across separate sessions for each of the subjects. At least 2 days but no more than two weeks intervened between drug testing sessions.

At each session, baseline diameter was first measured in darkness and light. Next, a single drop of saline, tropicamide, or dapiprazole was placed onto the lower limbus of the left eye by nursing or medical staff, and the subject was instructed to move his/her eye around to facilitate absorption. To verify stabilization of resting diameter in the treated eye, pupil diameters of both eyes were monitored at 5–10-minute intervals in light and darkness for approximately 25 minutes, at which time recording was initiated. Pilot testing had indicated that 23–25 minutes was optimal for effective blockade of the sphincter or dilator. Note that no attempt was made to provide absolute blockade through use of additional drops (as is often the case during clinical ophthalmological examinations) or to modify dosage for irises of different colors. Other than the expected effects of a possible brief sting when the drop was first placed in the eye, and reddening of the sclera after dapiprazole, there were no additional side effects reported by any of the subjects.

### 2.3. Cognitive Task Procedures

Subjects were seated in a darkened chamber. Head position was maintained using a head and chin rest. The stimulus was a focused red (680 nm) light-emitting diode (LED), 4 mm in diameter, placed 50 cm from the eye, subtending a visual angle of 0.39°. The LED produced a luminance of 4.80 cd/m^2^ at the eye. Three dim red LEDs masked to pinhead size surrounding the focused LED were used to provide a focal point, producing a barely detectable background luminance of no more than 0.03 cd/m^2^.

On each day of testing, 1 block of light stimuli was presented for each of three tasks. Blocks consisted of 11 light stimuli, with the light turned on for 1 sec, and off for 3 sec. After at least 2 min of dark adaptation, each of the three conditions described below was presented in randomized order. (1) In the No Task condition, the subject was instructed only to look at the red LED. (2) In the “Add 1” condition, the subject was told a randomly generated seed number by intercom and asked to slowly add 1 to that number, saying the result aloud, and continue to add 1 subsequently throughout the recording period. (3) In the “Subtract 7” condition, the subject was told a random seed number by intercom and was instructed to begin with that number and continue to subtract 7, reporting the result verbally as in the “Add 1” condition. Light stimuli in the latter two conditions were initiated after the subject began to report verbally. The verbal responses of the subjects were recorded but were used to verify that the subjects were attending to the task, and no modifications were made for intermittent errors in the Subtract 7 condition, which were few.

### 2.4. Pupillographic Recording

Pupil diameter was recorded using an ISCAN, Inc., Model RK-406 Pupillometer. An infrared light source permitted measurement of the pupil darkness. Accuracy of measurement with this system and optics is better than .025 mm. The analog output was digitized at 62.5 Hz (16 ms intersampling time) and stored on magnetic media during the entire recording epoch. When verbalizing the results of mathematic operations (in the Add 1 and Subtract 7 conditions), small head movements sometimes occurred due to mouth movements. A remote control system was used to adjust the camera angle to keep the eye within recording limits during head movements produced by verbalizations, and such movements were not sufficiently large to produce errors in pupil measurement.

### 2.5. Data Analysis

Offline, individual trial data were filtered using a 9.2 Hz two-pass digital filter and scaled to mm. Each trial was displayed on a video monitor, evaluated for blinks, and edited if necessary. The automatic editing algorithm attempted to define beginning and end points for blinks, which could be modified by the experimenter. A linear interpolation was then applied. Trials were excluded if blinks occurred during either the 1 sec period of stimulus presentation (since not all of the stimulus energy had been presented to the eyes) or during the period at which maximum constriction had possibly occurred (precluding determination of maximum constriction). As is typical in stimulating with a series of light stimuli [7], the first stimulus in each train of 11 produced a larger initial diameter and greater constriction than subsequent trials, since the period of prestimulus darkness was always greater. Subsequently, data for trial 1 were omitted from analyses.

The remaining artifact-free or corrected trials 2–11 for each condition were then averaged at each time point, beginning 200 msec prior to stimulus onset. From each average, amplitude and latency measures were automatically determined using locally designed software and algorithms [7]. Amplitude measurements included initial diameter prior to stimulus onset (the mean diameter over −200 to −120 msec); constricted diameter (the minimum diameter at the termination of constriction); and extent of constriction (initial diameter minus constricted diameter). Latencies were obtained for the times of the start of constriction and end of constriction. The maximum rate of constriction following the onset of constriction and maximum rate of redilation following the termination of constriction also were extracted.

Data were analyzed as a repeated measures design for the factors of drug (3 levels: placebo, dapiprazole, or tropicamide, or 2 levels when only placebo and dapiprazole were directly compared) × task condition (3 levels: No Task, Add 1, Subtract 7). Greenhouse-Geisser corrected probability levels are reported for ANOVAs where appropriate. Significance levels for post hoc *t*-tests among task conditions (No Task, Add 1, Subtract 7) were Bonferroni-corrected for multiple paired comparisons and are reported as significant only if *p* < 0.017.

## 3. Results

**Figure 1:**
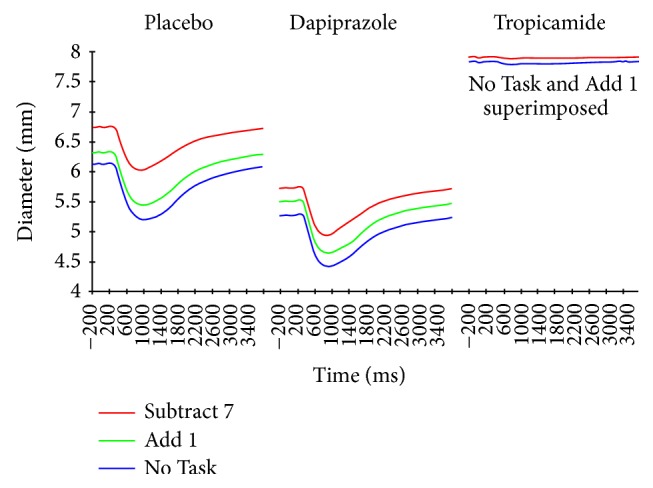
Average pupillary response to light stimuli 2–11 for 29 subjects. Columns represent separate placebo, dapiprazole, and tropicamide instillations. Conditions are No Task (blue line), Add 1 (green line), and Subtract 7 (red line). Note that the No Task and Add 1 conditions are completely superimposed for the tropicamide administration. Light stimulus duration was from 0 to 1000 msec.

Figure 1 presents averaged responses for each pharmacological recording condition by task demand. Mean values for all pupillary measures are presented in Table 1. Subjects made more verbal reports in the easier Add 1 condition than in the Subtract 7 condition. Following both the placebo and dapiprazole administrations, the pupil showed a typical constriction beginning at a mean of 346 msec after light onset. As expected, there was nearly total elimination of the light reaction by tropicamide. Overall initial diameter compared to placebo was decreased when the dilator muscle was blocked with dapiprazole and increased when the sphincter was blocked by tropicamide (*F*_1.4,36.6_ = 98.73, *p* < 0.001).

### 3.1. Prestimulus Diameter

Prestimulus initial pupil diameter was significantly increased with increasing task demand (Light Only < Add 1 < Subtract 7) for both the placebo (*F*_1.5,42.1_ = 23.04, *p* < 0.001) and dapiprazole (*F*_1.8,51.3_ = 18.73, *p* < 0.001) conditions, with significant linear trends related to task conditions (*p* ≤ 0.001) for both of these drug conditions. In the placebo condition, pupil diameter was significantly increased for the Subtract 7 condition as compared to both the No Task and the Add 1 conditions. All conditions differed significantly from each other in the dapiprazole condition. In contrast, during tropicamide administration, there was a significant increase for the Subtract 7 condition over both the No Task and Add 1 conditions (*F*_1.6,45.2_ = 8.58, *p* < 0.001), but no difference between the No Task and Add 1 conditions (Figure 1).

### 3.2. Light Reaction Amplitude

**Figure 2:**
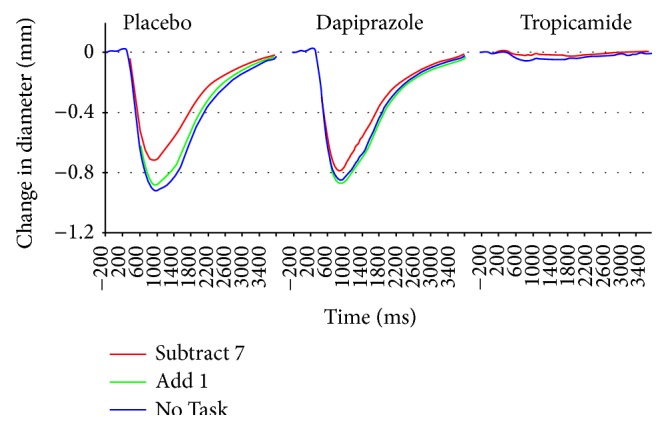
Same data as Figure 1, with initial diameters subtracted and scale increased. Note reduction of the light reaction for the Subtract 7 condition in both the placebo and dapiprazole conditions. For the dapiprazole administration, the data for the No Task and Add 1 conditions are nearly identical and are completely superimposed as a single line in the tropicamide condition with No Task condition overlaying the Add 1 data completely.

To illustrate effects of task demand on the light reaction, the data of Figure 1 are replotted after subtraction of initial diameter in Figure 2. No consistently detectable constriction amplitude was measurable in most individual subjects after administration of tropicamide. The average response across subjects was still characterized by a very small constriction; the dose of tropicamide employed is unlikely to have produced complete elimination of sphincter activity in all subjects. (Values of constriction amplitude and latencies for the tropicamide condition in Table 1 were extracted from the across subject average waveforms.)

Statistical analyses of light reaction parameters were computed from the data of the placebo and dapiprazole conditions. When the amplitude of the light reaction was measured, there was a significant main effect of task (*F*_1.7,46.8_ = 12.68, *p* < 0.001). The light reaction amplitude was significantly reduced in the Subtract 7 condition relative to the No Task and Add 1 conditions.

For comparison, changes in the amplitude of the light reaction also are expressed as percentages in Table 1. Note, however, that percent change tends to exaggerate the effects of modulation of pupil diameter, since the same absolute change for a small pupil is proportionally greater than for a larger initial diameter. Only absolute change was utilized for our analyses.

There was also a significant interaction of task by drug (placebo versus dapiprazole) condition (*F*_1.9,53.8_ = 4.31, *p* = 0.02). This was associated with a larger constriction in the placebo than dapiprazole response in the No Task condition, which may reflect a minor floor effect for the dapiprazole condition; in contrast, a larger constriction was observed in the dapiprazole than placebo response for the Subtract 7 condition (see Figure 2; data in Table 1).

### 3.3. Latency Analyses

Latency analyses were restricted to the data for the placebo and dapiprazole conditions (values extracted from the group mean averages are presented in Table 1 for beginning and end of constriction in the tropicamide condition). There were no significant differences in latency to start of constriction among task conditions or after dapiprazole administration (mean 345.7 msec; all *F* values <1.9 for main effects and interactions). Similarly, the latency to reach maximal rate of constriction (410.6 msec) did not differ among conditions.

The end of constriction occurred earlier using dapiprazole compared to placebo (*F*_1,25_ = 10.18, *p* = 0.004). There was a main effect of task, with earlier latencies for higher processing loads (Subtract 7 < Add 1 < No Task; *F*_1.9,53.8_ = 12.52, *p* < 0.001), but no significant interaction of task by drug effect. There also was a main effect of task in the time to redilation of the pupil to 50% of initial diameter following the end of constriction (Subtract 7 < Add 1 < No Task; *F*_1.9,53.8_ = 12.52, *p* < 0.001); this effect became nonsignificant after covarying the time to end of constriction.

## 4. Discussion

Inhibition of the pupillary light reaction by administration of a demanding cognitive task occurred under placebo (replicating findings for a no-drug condition reported by Steinhauer et al. [11]), and during blockade of the pupillary dilator muscle by dapiprazole. This indicates that the primary pathway for reduction of the light reaction was mediated by parasympathetic inhibitory processes at the Edinger-Westphal complex of the oculomotor nucleus (n. III). This parallels findings for the fear-inhibited light reflex when dapiprazole was used by Giakoumaki et al. [15]. Changes in overall diameter with increasing task demand were present in both the placebo and dapiprazole conditions, indicating that even slight increases in task demand which were not sufficient to produce inhibition of the light reaction (i.e., Add 1) were also mediated primarily at this parasympathetic center. In contrast, when the pupillary sphincter was blocked by tropicamide, the resulting sympathetic response showed only a small, though significant, overall increase in diameter for the high demand condition, but no difference between No Task and low demand conditions. The extent of the sympathetic contribution to overall diameter during the difficult task may have been limited by ceiling effects.

Overall diameter was increased by the easy task operation (Add 1) and increased further by the more demanding task (Subtract 7). We have suggested that even slight increments in diameter associated with minimal task requirements are likely attributable to increasing activity of the reticular activating system [11], which is one of the key inhibitory inputs to the Edinger-Westphal complex [3]. It is possible that cortical activation during task performance also contributes to inhibition associated with overall diameter. Only a small proportion of task-induced increase in diameter was related to stimulation of the dilator muscles by the sympathetic pathway, which was isolated when tropicamide was used to block activity of the sphincter muscles.

A separate component leading to inhibition at the oculomotor center appears to be activated when cognitive difficulty is invoked. Inhibition of the light reaction was obtained only under conditions of the demanding Subtract 7 task. This decrement was clearly observed when the dilator was blocked, indicating that all of the effective inhibitory activity occurs at the Edinger-Westphal complex. This interpretation is consistent with the report of Heller et al. [17] who examined the light reaction (without any task requirement) while using thymoxamine, which also blocks the alpha adrenergic receptors of the dilator. Administration either of pentagastrin or the cold pressor test, which both evoke subjective anxiety associated with sympathetic arousal, increases pupil diameter but does not inhibit the amplitude of the light reflex [18]. Given that the inhibition was specific to the difficult task, it is not likely to be associated with the verbalization requirement of the Add 1 and Subtract 7 conditions. We suggest that the origin of this inhibition is likely to be modulated by cortical regions that are active during complex processing.

Lowenstein [19] described frontal cortical descending pathways with both direct and indirect (via thalamic-hypothalamic) projections that have inhibitory effects on the Edinger-Westphal nuclei. The decrease in light reaction is attributed to activity of these pathways. Increasing consideration has been paid to the possible contributions of locus coeruleus activity that may contribute to modulation of both tonic and phasic pupillary changes [20–22]. However, the exact pathway of these contributions is not entirely clear in human. For example, the direct inhibitory connections established in lower mammals such as rat and cat have not been found in humans, though correlations of locus coeruleus activity and patterns of pupillary change have been seen in monkey [20].

As in the case of increases in overall diameter, it is not possible in this design to preclude involvement of reticular inhibitory activity contributing to the decreased light reaction amplitude. However, it is clear that more than one process is contributing to the patterns of activity influencing parasympathetic outflow, as changes in diameter are easily stimulated, but inhibition of light reaction components is more difficult to produce.

The association of increasing diameter with decreased light reaction amplitude suggests that these effects are not range limited, since a larger diameter should allow for a more extensive constriction amplitude if the response is primarily driven by available range of movement. A corresponding observation has been made for studies of the light reaction in patients with anxiety disorders, in which overall diameter is increased while light reaction amplitude is decreased [5]. When diazepam is administered to reduce anxiety, there is both a decrease in overall diameter and an increase in light reaction amplitude [4].

An indication of peripheral interactions is suggested by the interaction of response amplitude by condition in the placebo and dapiprazole conditions. As can be seen in Figure 2, there is actually a larger constriction response to Subtract 7 under dapiprazole than placebo, even though the initial diameter is greatly decreased under dapiprazole. The fact that antagonism of the dilator muscles is not present in this condition may account for the relative enhancement of the contraction.

The use of separate pharmacological agents to isolate either the sphincter or dilator muscles by blocking the respective cholinergic or adrenergic receptor sites was successful at discriminating different processes during this paradigm. One advantage of this approach is that there was no interference with central sympathetic or parasympathetic activity, as would be the case with centrally acting pharmacological agents. Similarly, the extent to which reciprocal inhibition may have occurred—the decrease of activity in one branch of the autonomic system when the other is actively stimulated [3, 23, 24]—would not be affected with this approach.

Results of the present study reinforce the earlier conclusion that ongoing task difficulty and cognitive operations influence not only overall pupil diameter, but also characteristics of the reflex reaction to light [11]. In particular, the difficulty of the operations involved has a directly quantifiable effect on the extent of constriction to light. In many instances of physiological activity, obtaining a pure baseline measure of activity is difficult. For the pupillary system, resting diameter tends to vary when no specific task is involved [3]. By using the response to a light as a base measure, the inhibition of the response provides a direct indication of relative increases in central activities that converge upon the E-W complex of the oculomotor nucleus. Consequently, tasks that presumably differ in cognitive complexity and difficulty can be titrated precisely when the light reaction can be introduced during performance of those tasks. A key goal for future studies is to determine the precise origins of the separate inhibitory inputs that modulate the light reaction during cognition.

## Figures and Tables

**Table 1:** Summary of pupillary measures [mean (standard deviation)] for placebo, dapiprazole, and tropicamide sessions as a function of task condition.

	Placebo	Dapiprazole	Tropicamide
		(block dilator)	(block sphincter)
Initial diameter (mm)			
No Task	6.122 (1.121)	5.268 (1.211)	7.842 (1.028)
Add 1	6.321 (1.176)	5.507 (1.215)	7.836 (0.989)
Subtract 7	6.742 (1.101)	5.727 (1.068)	7.912 (0.999)
Constriction amplitude (mm)			
No Task	0.956 (0.460)	0.867 (0.365)	0.021^*∗*^
Add 1	0.903 (0.475)	0.885 (0.436)	0.048^*∗*^
Subtract 7	0.731 (0.446)	0.799 (0.409)	0.060^*∗*^
% Change (constriction/initial diameter × 100)			
No Task	15.62%^*∗*^	16.46%^*∗*^	0.27%^*∗*^
Add 1	14.29%^*∗*^	16.07%^*∗*^	0.61%^*∗*^
Subtract 7	10.84%^*∗*^	13.95%^*∗*^	0.76%^*∗*^
Start of constriction (msec)			
No Task	342.2 (42.5)	343.4 (38.5)	424^*∗*^
Add 1	351.4 (38.7)	337.2 (36.0)	408^*∗*^
Subtract 7	355.1 (38.3)	345.2 (30.7)	360^*∗*^
End of constriction (msec)			
No Task	1048.0 (182.4)	952.0 (152.7)	808^*∗*^
Add 1	1018.5 (204.4)	918.2 (97.8)	816^*∗*^
Subtract 7	1099.1 (104.8)	872.6 (70.9)	824^*∗*^
Latency to 50% recovery of initial diameter (msec)			
No Task	1872.83 (527.2)	1788.41 (201.2)	—
Add 1	1703.45 (598.6)	1690.21 (438.1)	—
Subtract 7	1608.0 (559.3)	1599.72 (421.3)	—

^*∗*^Values obtained from grand means across subjects.

—: no data available as changes too small to assess.
